# Enhanced biofilm formation in dual-species culture of *Listeria monocytogenes* and *Ralstonia insidiosa*

**DOI:** 10.3934/microbiol.2017.4.774

**Published:** 2017-09-11

**Authors:** Yunfeng Xu, Attila Nagy, Gary R. Bauchan, Xiaodong Xia, Xiangwu Nou

**Affiliations:** 1 College of Food Science and Engineering, Northwest A&F University, Yangling, Shaanxi 712100, China; 2 USDA Agricultural Research Service, Beltsville Agricultural Research Center, Beltsville, Maryland 20705, USA

**Keywords:** biofilm, *Listeria monocytogenes*, *Ralstonia insidiosa*

## Abstract

In the natural environments microorganisms coexist in communities as biofilms. Since foodborne pathogens have varying abilities to form biofilms, investigation of bacterial interactions in biofilm formation may enhance our understanding of the persistence of these foodborne pathogens in the environment. Thus the objective of this study was to investigate the interactions between *Listeria monocytogenes* and *Ralstonia insidiosa* in dual species biofilms. Biofilm development after 24 h was measured using crystal violet in 96-well microtiter plate. Scanning electron microscopy and cell enumeration were employed after growth on stainless steel coupons. When compared with their single species counterparts, the dual species biofilms exhibited a significant increase in biofilm biomass. The number of *L. monocytogenes* in co-culture biofilms on stainless steel also increased significantly. However, there was no effect on the biofilm formation of *L. monocytogenes* when cultured with *R. insidiosa* separated by a semi-permeable membrane-linked compartment or cultured in *R. insidiosa* cell-free supernatant, indicating that direct cell-cell contact is critical for this interaction.

## 1. Introduction

Biofilms are living microbial communities attached to a solid surface or flowing in aqueous systems. Mature biofilms are highly organized ecosystems in which microbial cells are embedded in extracellular matrices with water channels that provide passages for the exchange of nutrients, metabolites and waste products [1]. Such biofilm structures can confer protection to bacterial cells and decrease the efficiency of cleaning and disinfection procedures [2]. Multispecies biofilms are the predominant forms of presence for microorganisms in the natural environments, and play important roles in the survival and persistence of microorganisms, including foodborne pathogens [3]. In such microbial communities, microorganisms interact in various ways that can be described as competition, antagonism, or synergism [4].

*Listeria monocytogenes* is a Gram-positive foodborne pathogen that is responsible for serious infections in immunocompromised individuals and pregnant women [5]. It is frequently found in various food processing environments and has been isolated from a range of food products including meat, milk, cheese, and vegetables [6]–[9]. Over the last few decades, *L. monocytogenes* continued to be a major public health threat [10]. A recent large scale outbreak was caused by contaminated cantaloupes grown and packed in one farm in Colorado which caused 147 infections and at least 33 deaths in the USA [11]. Biofilm formation has been suggested an important factor for the persistence of *L. monocytogenes* in the environment and the contaminations of food processing facilities. However, *L. monocytogenes* cultivated alone in classic laboratory media, such as tryptone soy broth (TSB) or brain-heart infusion (BHI) broth, exhibit relatively low potential for forming biofilm [12],[13].

*Ralstonia insidiosa* is a Gram-negative, nonsporulating, aerobic, nonfermentative, motile rod. It has been isolated from the respiratory secretions of cystic fibrosis patients, river and pond water, soil, activated sludge [14] and has also been detected in water distribution systems [15]. *R. insidiosa* FC 1138 was isolated from a fresh-cut produce processing plant and has been shown a strong biofilm producer. When co-cultured with *R. insidiosa*, the presence *E. coli* O157:H7 in the biofilms significantly increase under various tested conditions [16]. In this study we evaluated the interactions of *L. monocytogenes* and *R. insidiosa* in biofilm formation during 24 h co-incubation. At this time period, although the biofilm is still considered in early stage, the interspecies interactions can be readily discerned.

## 2. Materials and Methods

### 2.1. Bacteria stains and growth conditions

*L. monocytogenes* and *R. insidiosa* strains (Table 1) originally isolated from various food and other sources were from our laboratory collections. They were maintained at –80 °C in TSB (BD, Franklin Lakes, NJ) with 25% glycerol. Before all experiments, frozen cells were subcultured on tryptic soy agar plates (TSA; BD) for 24 h, and then one colony was transferred into TSB and grown to stationary phase. *L. monocytogenes* strain FS2025 was used as a representative in experiments of biofilm formation on stainless steel surface and in compartmentalized cultures.

### 2.2. Biomass quantification

A static biofilm formation assay was performed in 96-well polystyrene plates (BD) as previously reported [17] with some modifications. Briefly, cells were cultured overnight in TSB. They were centrifuged at 4,500 g and re-suspended in 10% TSB. The OD_600_ value was adjusted to 0.5 and then diluted 100 fold in 10% TSB. Aliquots of 200 µL of this inoculated culture, containing either a single strain or a mixture of *R. insidiosa* and *L. monocytogenes*, were transferred to individual wells for biofilm formation. The plates were incubated at 30 °C for 24 h without shaking. After removal of the liquid culture, attached biofilms were stained with crystal violet. Excessive dye was removed and rinsed three times with PBS. The retained dye was dissolved in 33% acetic acid, and absorbance was measured at 570 nm to quantify total biomass.

**Table1.** Bacterial strains used in this study.

Strain	Serotype	Isolation Source	Collections
*R. insidiosa*			
FC1138	NA	Fresh produce facility	EMFSL
*L. monocytogenes*			
FS2005	1/2a	Milk	EMFSL
FS2006	3b	Milk	EMFSL
FS2007	1/2b	Milk	EMFSL
FS2008	4b	Milk	EMFSL
FS2009	4b	Spinal fluid of child with meningitis	ATCC
FS2017	4b	Beef and pork sausage	EMFSL
FS2018	1/2b	Hard salami	EMFSL
FS2019	1/2a	Frankfurter	EMFSL
FS2025	1/2b	Cantaloupe	NRRL
FS2026	1/2a	Cantaloupe	NRRL

EMFSL: Environmental Microbial and Food Safety Laboratory, USDA ARS; ATCC: American Type Culture Collections; NRRL: Northern Regional Research Laboratory, USDA ARS.

### 2.3. Compartmentalized cultures

Cell culture plates (12-well) and matching insert compartments with 0.4 µm pore size polycarbonate membrane (Corning, NY, USA) that supported free diffusion of culture medium and metabolites were used to physically separate *R. insidiosa* and *L. monocytogenes* strains during biofilm formation. A bacterial suspension (1.5 ml in 10% TSB) of *L. monocytogenes* strain was added to each well of the plate, and an equal volume of the same medium with or without inoculum of *R. insidiosa* was added in the insert compartment on top of the wells. After incubation at 30 °C for 24 h, the inserts were removed. Biofilms in the base compartments were determined by crystal violet stain. A reciprocal format with *L. monocytogenes* in the insert compartment was similarly tested.

### 2.4. Scanning electron microscope (SEM) of biofilms

Type 304 stainless steel coupons with #4 brush finishing (30 mm × 15 mm × 2 mm, Remaly Manufacturing, Tamaqua, PA) were used for biofilm formation. Coupons were placed in a sterile deep-well micro-dilution block with 2 ml of bacteria suspension in each well, which allows the submersion of half of the coupon. They were incubated at 30 °C for 24 h without shaking. Then the coupons were washed three times with PBS and fixed with formaldehyde for 1 h. Biofilms structures were observed using a Hitachi S-4700 low temperature scanning electron microscope (Hitachi High Technologies America, Inc., Schaumburg, IL) after coating with platinum.

### 2.5. Bacterial enumeration

Enumeration of bacteria in biofilms formed on stainless steel coupons (75 mm × 25 mm × 2 mm, Remaly Manufacturing) was carried out as follows: The coupon was half submerged in 20 ml growth medium in a 50 ml Falcon tube and allowed static incubation at 30 °C for 24 h. The coupons were rinsed three times with 3 ml of sterile PBS. Cells remaining attached to the coupon surface were harvested by uni-directional scraping with a sterile cotton-headed stick and released into 10 ml sterile PBS by rigorous twirling and squeezing against vial side. The resulted samples were then vortexed vigorously for 1 min to facilitate cell dispersion. Cell suspension was 10-fold serially diluted and plated on TSA containing 1 g/L of 5-Bromo-4-chloro-3-indolyl β-D-glucopyranoside for enumerating *L. monocytogenes* (blue colonies) and *R. insidiosa* (white colonies).

### 2.6. Statistical analysis

A one-way analysis of variance (ANOVA) was performed for statistical analysis using the SPSS software version 19.0. Data were presented as the mean values ± SD (n = 3). Differences were considered significant when *P* < 0.05.

## 3. Results and Discussion

### 3.1. Enhanced biofilm formation in mixed culture

**Figure 1.**
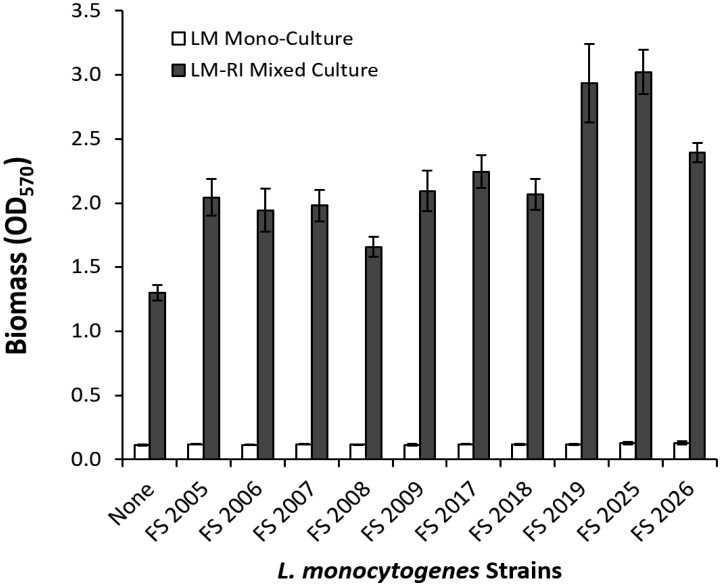
Biofilm formation of *L. monocytogenes* strains in monocultures and in mixed cultures with *R. insidiosa* measured by the crystal violet staining assay.

Biofilm formation by 10 *L. monocytogenes* strains of varying serotypes and isolation origins were examined as monoculture and in mixed culture with *R. insidiosa* strain FC1138 (Figure 1). When cultured alone, each of the *L. monocytogenes* strains exhibited weak ability of biofilm formation in 10% TSB, as indicated by the minimal biomass accumulation. No difference was observed among the tested *L. monocytogenes* strains. Previous studies by Harvey et al [12] showed that *L. monocytogenes* strains had varying biofilm formations, although the majority was weak biofilm producers. This was also consistent with observations in our previous study [18] which showed that all *L. monocytogenes* strains tested were weak in biofilm formation in monocultures. In contrast, biomass accumulation in the mixed cultures of *R. insidiosa* with individual *L. monocytogenes* strains was significantly higher, indicating strong biofilm formation. Since *R. insidiosa* alone is a strong biofilm former, it could be expected that the high biomass accumulation was due to the strong biofilm formation by *R. insidiosa*. However, the biomass accumulation in the mixed culture greatly exceeded the sum of the two individual strains, indicating a synergistic interaction between *L. monocytogenes* and *R. insidiosa* in forming biofilms. This synergistic interaction was also previously observed between *R. insidiosa* and *E. coli* O157:H7 strain [18].

### 3.2. Cell contact dependent interactions

**Figure 2.**
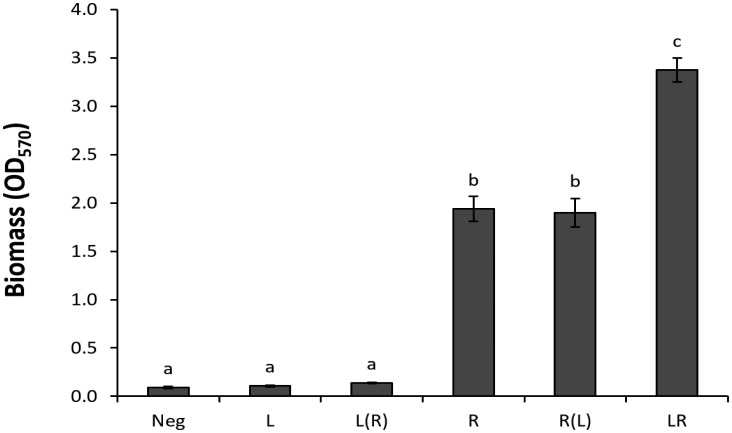
Biofilm formation of *L. monocytogenes* and *R. insidiosa* in physically separated cultures. Biofilm formation was measured by crystal violet staining of biomass in the base compartment. Letter in the parentheses indicate *Listeria* or *Ralstonia* cells in the insert compartment. Neg: Negative control, L: biofilm formation of *L. monocytogenes*, L(R): effect of *R. insidiosa* (gown in the insert compartment) on biofilm formation of *L. monocytogenes* (Grown in the base compartment), R: biofilm formation of *R. insidiosa*, R(L): effect of *L. monocytogenes* (Insert compartment) on biofilm formation of *R. insidiosa* (Base compartment), LR: dual-species biofilm formation in mixed culture in the base compartment.

Quorum sensing is a common mechanism of intra- and inter-species communications in microbial communities. This mechanism relies on small diffusible molecules, such as acy-homoserine lactone (AHL), oligopeptides, and autoinducer 2, as signal molecules [19]. To examine the possibility that the synergistic interaction in biofilm formation was affected by a mechanism akin to quorum sensing, *R. insidiosa* and *L. monocytogenes* cells were inoculated in two separate compartments linked through 0.4 µm pore size filter membrane that supported free diffusion of culture medium and metabolites. Biomass production were determined (Figure 2) after incubation at 30 °C for 24 h. No significant difference was observed in the *L. monocytogenes* monoculture biofilms formed with or without the presence of *R. insidiosa* in a semi-permeable membrane-linked compartment. This observation does not support the notion that *R. insidiosa* metabolites or secreted signal molecules play significant roles in promoting the incorporation of *listeria* strains into dual species biofilms. In a reciprocal setting, *R. insidiosa* biofilm formation was not affected by the presence of *L. monocytogenes* in a connected semipermeable compartment. We also examined the biofilm formation of *L. monocytogenes* in the presence or absence of potential *R. insidiosa* diffusible signal molecules by supernatant replacement. Replacing supernatant from *L. monocytogenes* overnight culture with that from *R. insidiosa* growth did not increase the biofilm formation by *L. monocytogenes* (Data not shown). Taken together, these observations indicated that the synergism of these two species in biofilm formation was dependent on direct cell to cell contacts. However, these observations do not preclude the possibility that diffusible signal molecules are also required for this synergistic interaction.

### 3.3. Biofilm formation on stainless steel and SEM observations

Polystyrene microplates and glasses are the most commonly used substrata for biofilm studies. However, these types of surfaces are scarcely used for food processing. We have previously shown that *R. insidiosa* promoted the incorporation of *E. coli* O157:H7 cells into dual species biofilms, using substrata including microplates, glass slides, and glass bottomed petri dishes to facilitate biofilm quantification and microscopic observations [16],[20]. In this study, we examined the synergism between *L. monocytogenes* and *R. insidiosa* in biofilm formation on stainless steel, which is the surface that is most commonly encountered in food processing environments. When *L. monocytogenes* and *R. insidiosa* were gown in 10% TSB and allowed to form biofilms on stainless steel coupons, visual inspection indicated that *L. monocytogenes* had minimal biofilm formation in monoculture, and that *R. insidiosa* formed thick biofilms both in monoculture and in the mixed culture with *L. monocytogenes*. These observations are similar to those made when the mono- and mixed cultures were grown in tissue culture plates, suggesting the difference in these substrata might not be critical for biofilm formation. After three consecutive rinses, cells remaining attached to the SS coupons were recovered and enumerated (Table 2). While comparable populations of *R. insidiosa* cells (∼8 log CFU/coupon) were recovered for both the mono and mixed cultures, *L. monocytogenes* cells recovered from the coupons in mixed culture (7.3 log CFU/coupon) were nearly 1.9 logs higher than those from the monoculture (5.4 log CFU/coupon). This nearly 100-fold differential in *L. monocytogenes* from the mono- and dual-species biofilms indicated that *R. insidiosa* strongly promoted the incorporation of *L. monocytogenes* cells into the dual species biofilms.

**Table 2.** Cell counts of bacteria in mono-culture and dual-species biofilms grown on stainless steel surface (log CFU/coupon).

Biofilm	*L. monocytogenes*	*R. insidiosa*
Mono culture	5.42 ± 0.31^a^	8.12 ± 0.05^c^
Mixed culture	7.32 ± 0.11^b^	7.99 ± 0.15^c^

The data represent the means and standard deviations of three independent experiments. Values followed by different letters in superscript are significantly different (*P* < 0.05).

**Figure 3.**
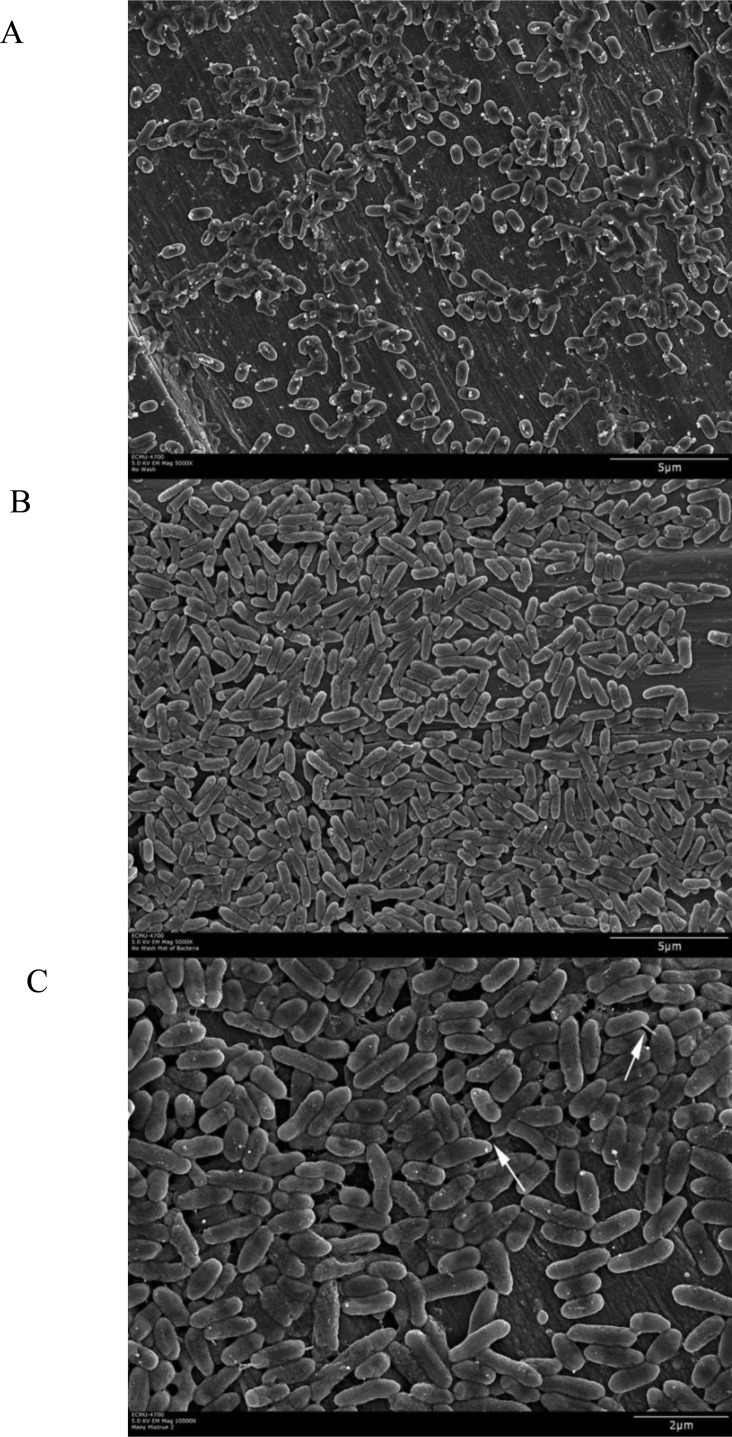
SEM observations of biofilm structure on stainless steel coupons. (A) *L. monocytogenes*, (B) *R. insidiosa*, and (C) dual-species. Arrows points to the observed short EPS filaments in the biofilms of the mixed culture.

The mono- and mixed culture biofilms formed on stainless steel coupons were directly visualized using scanning electron microscopy (SEM) after rinsing and fixation (Figure 3). Consistent with above enumeration, *L. monocytogenes* cells in pure culture sparsely attached to the stainless steel surface without forming continuous biofilms (Figure 3A). In contrast, the SS coupons from *R. insidiosa* mono- and the mixed cultures were colonized by bacterial cells at much higher density which exhibited continuous biofilm characteristics (Figure 3B and Figure 3C). We have been unable to distinguish *L. monocytogenes* cells from those of *R. insidiosa* in the mixed culture biofilms by cell morphology under SEM, although it seemed the cells of these two species had slight differences in cell size and shapes, and the cells in the mixed culture biofilms seemed more heterologous. In our previous studies involving biofilms formed on glass fiber filter paper using SEM, *R. insidiosa* exhibited extensive web-like networking of extra-cellular polymeric substances (EPS), to which *E. coli* O157:H7 cells seemed to interact. In the current study of biofilm formation on stainless steel surface, such extensive EPS networking was not observed. Nevertheless, cells in the mixed culture biofilms were frequently observed with short EPS filaments (arrows in Figure 3C) connected to neighboring cells. Such cell connecting EPS filaments were rarely observed with the cells from the *R. insidiosa* monoculture biofilms, and their importance in cellular interactions is not clear. Dubey and Ben-Yehuda [21] hypothesized that such filaments play critical roles in intraspecies communications.

## 4. Conclusions

Overall, we demonstrated that co-culturing *L. monocytogenes* and *R. insidiosa* significantly enhanced biofilm formation. *L. monocytogenes* cells incorporated into dual-species biofilms formed on stainless steel surface increased by nearly 100-fold compared to monoculture. This synergistic interaction in biofilm formation was dependent upon cell-cell contact. The nature of the interaction has yet to be determined.
